# Ameliorating the drought stress tolerance of a susceptible soybean cultivar, MAUS 2 through dual inoculation with selected rhizobia and AM fungus

**DOI:** 10.1186/s40694-023-00157-y

**Published:** 2023-05-03

**Authors:** Revanna Ashwin, Davis Joseph Bagyaraj, Basavaiah Mohan Raju

**Affiliations:** 1 Centre for Natural Biological Resources and Community Development (CNBRCD), 41 RBI Colony, Anand Nagar, Bangalore, Karnataka 560024 India; 2 Centre for Research and Development (CRD), PRIST University, Vallam, Thanjavur, Tamil Nadu 613403 India; 3 Department of Crop Physiology, University of Agricultural Sciences, Bangalore, Karnataka 560065 India

**Keywords:** AM fungi, *Ambispora leptoticha*, *Bradyrhizobium liaoningense*, Dual inoculation

## Abstract

**Background:**

Drought stress is currently the primary abiotic stress factor for crop loss worldwide. Although drought stress reduces the crop yield significantly, species and genotypes differ in their stress response; some tolerate the stress effect while others not. In several systems, it has been shown that, some of the beneficial soil microbes ameliorate the stress effect and thereby, minimizing yield losses under stress conditions. Realizing the importance of beneficial soil microbes, a field experiment was conducted to study the effect of selected microbial inoculants namely, N-fixing bacteria, *Bradyrhizobium liaoningense* and P-supplying arbuscular mycorrhizal fungus, *Ambispora leptoticha* on growth and performance of a drought susceptible and high yielding soybean cultivar, MAUS 2 under drought condition.

**Results:**

Drought stress imposed during flowering and pod filling stages showed that, dual inoculation consisting of *B. liaoningense* and *A. leptoticha* improved the physiological and biometric characteristics including nutrient uptake and yield under drought conditions. Inoculated plants showed an increased number of pods and pod weight per plant by 19% and 34% respectively, while the number of seeds and seed weight per plant increased by 17% and 32% respectively over un-inoculated plants under drought stress condition. Further, the inoculated plants showed higher chlorophyll and osmolyte content, higher detoxifying enzyme activity, and higher cell viability because of less membrane damage compared to un-inoculated plants under stress condition. In addition, they also showed higher water use efficiency coupled with more nutrients accumulation besides exhibiting higher load of beneficial microbes.

**Conclusion:**

Dual inoculation of soybean plants with beneficial microbes would alleviate the drought stress effects, thereby allowing normal plants’ growth under stress condition. The study therefore, infers that AM fungal and rhizobia inoculation seems to be necessary when soybean is to be cultivated under drought or water limiting conditions.

**Supplementary Information:**

The online version contains supplementary material available at 10.1186/s40694-023-00157-y.

## Background

The world's population is estimated to reach 10 billion by 2050 demanding additional 50% increase in food requirement [1]. The increased demand for food presents a significant challenge for the global food system, including increasing agricultural productivity and minimizing yield losses in major food crops. One of the factors posing a primary challenge to world’s food productivity is abiotic stress and more specifically, the drought stress which causes substantial yield loss in many food crops globally [2, 3]. Soybean [*Glycine max* (L.) *Merrill*], one of the world’s fastest growing protein-rich oilseed crops also suffers from drought stress causing a significant yield loss due to unprecedented rainfall and poor management. In India, the productivity is only about 25% of its potentiality as it is mainly grown as a rain-fed crop experiencing erratic, uneven and inadequate rainfall leading to a low productivity compared to other countries [4]. Besides water scarcity, nutrient deficiency and low nutrient use efficiency of the crop also causes low productivity of soybean [5] which is more sensitive to water deficits during the flowering, pod set and pod filling stages [6]. Therefore, in this scenario of unprecedented rainfall, there is a need to develop suitable cultivars tolerant to moisture stress or develop strategies to improve the stress tolerance of the existing cultivars through possible interventions [7, 8].

Beneficial soil microbes have been shown to offer several benefits including conferring drought and other abiotic stress tolerance to the host plants [9]. Microbial interactions with the plants are an integral part of the living ecosystem. They are natural partners modulating local and systemic mechanisms in plants offering defence under different developmental stages of a plant at molecular, physiological and biochemical level under various external stress conditions [10]. Symbiotic association of plants with certain beneficial microorganisms can enhance growth in host plants by mitigating the stress effects and providing profound benefits to the crop plants by enhancing the plant’s growth rate, stimulating the production of phyto-hormones, siderophores, etc. along with up-regulating the expression of dehydration response and antioxidant genes during abiotic stresses [9, 10].

Arbuscular mycorrhizal (AM) fungi are a type of beneficial fungi that form a symbiotic relationship with the roots of most vascular plants. They colonize the host roots and facilitate the plants to obtain nutrients and water from the soil and in-turn, the host plant provides the fungi with the required carbohydrates. Many studies have demonstrated that, AM fungal association helps to maintain bio-geochemical cycling even under drought conditions which play a critical role in ecosystem resilience [11]. Another group of symbiotic bacteria i.e. rhizobia accounts for 97% of the N fixation of the total plant N requirement [12, 13]. AM fungi when co-inoculated with bradyrhizobia may directly and preferentially stimulate rhizobial nodule function and have a more substantial impact on enhancing drought tolerance compared to rhizobia alone since AM fungi improve the plant’s growth through enhanced water and P uptake from soil, osmotic adjustment in roots, high leaf water potential, and reduced oxidative damage to lipids [14–16]. Although a large amount of information is available regarding the beneficial effects of microbes, the knowledge regarding the ‘on-field response’ of plants to simultaneous exposure to multiple stresses at different growth stages and the role of beneficial soil microbes in mitigating the stress effects is very scanty.

Literature survey suggests that, there needs to be more interaction studies, particularly under field conditions between rhizobia and AM fungi in soybean for alleviating the moisture stress effects [13, 17, 18]. A micro-plot experiment was conducted earlier addressing this gap using  a selected microbial consortium on the drought-susceptible cultivar, MAUS 2 and the drought-tolerant cultivar, DSR 12. Drought stress was imposed for 20 days during the flowering stage and the results indicate that, dual inoculation significantly improved the plant's growth, nutrient uptake and yield of both the cultivars under drought stress conditions [19]. To validate these results, a field experiment was conducted using  a susceptible cultivar, MAUS 2 and the drought stress was imposed at two different growth stages namely, flowering and pod filling (hereafter, these two developmental stages are referred to as “both the stress periods”). The present study provides more insight and understanding about the role of bradyrhizobia sp. and AM fungus in conferring drought stress tolerance at multiple growth and developmental stages to the susceptible soybean cultivar, MAUS 2 under field conditions.

## Results and discussion

**Fig. 1:**
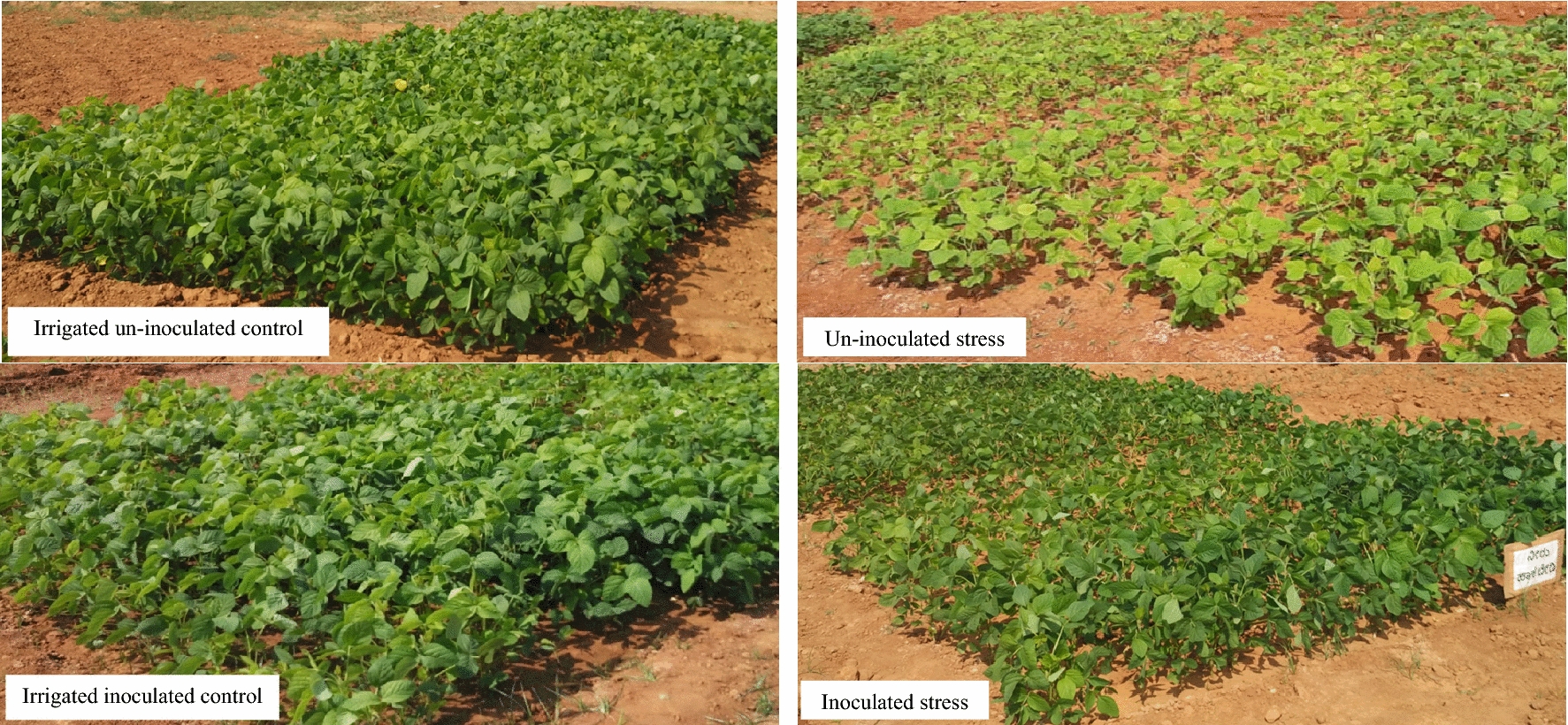
Response of soybean cultivar, MAUS 2 inoculated with *Ambispora leptoticha* + *Bradyrhizobium liaoningense* grown under irrigated and moisture stressed field conditions

The information on the field response of soybean plants exposed to moisture stress at different growth stages is very scanty. This investigation was  attempted to understand how soybean plants respond to moisture stress at different developmental stages. Earlier workers have reported that, soybean crop is more sensitive to moisture stress at flowering and pod filling stage, causing significant yield loss [6]. However, the present field study conducted to examine the role of dual inoculation comprising of N-fixing root nodulating bacteria, *Bradyrhizobium liaoningense* and P-supplying AM fungi, *Ambispora leptoticha* in ameliorating the moisture stress effects at multiple growth and developmental stages in a susceptible soybean cultivar, MAUS 2 showed a positive response (Fig. 1).

*Assessing the soil moisture status to confirm the drought stress*: Analysis of soil samples collected during both the stress periods indicate that, the soil moisture content was higher in irrigated plots with a moisture content of 14% compared to 7% in stressed plots. Interestingly, in the stressed plots, the soil moisture content was significantly higher in inoculated than un-inoculated plots suggesting that the dual inoculation improved the soil water retention capacity. However, under irrigated conditions, soil moisture content was not significantly different between inoculated and un-inoculated plots. Soil analysis recorded a water potential of -3.3 bars and -10 bars in irrigated control and stressed plots respectively (Additional file 1: Fig. S1) suggesting that, the plants in the stressed plots indeed experienced the moisture stress effects. However, no significant difference was observed between inoculated and un-inoculated plots in soil water potential at both the stress periods.

**Fig. 2:**
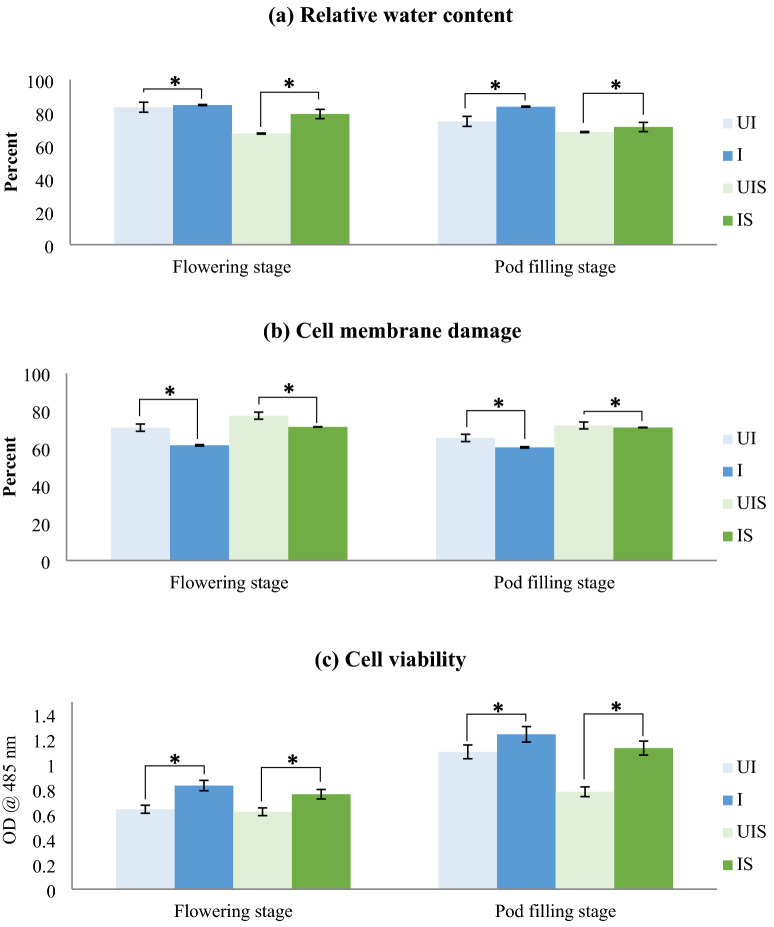
Influence of dual inoculation on **a** Relative water content, **b** Cell membrane damage and **c** Cell viability of leaves analyzed at flowering and pod filling stage of a drought susceptible soybean cultivar, MAUS 2 grown under irrigated and moisture stressed field conditions. Dual inoculation: *Ambispora leptoticha* + *Bradyrhizobium liaoningense*; *UI* un-inoculated, *I* inoculated, *UIS* un-inoculated stress, *IS* inoculated stress, Flowering stage: 1st stress period (35–60 DAS), Pod filling stage: 2nd stress period (85–100 DAS); Significant differences (p ≤ 0.05) relative to controls UI & UIS to their respective treatments I & IS are indicated by asterisk (*)

*Influence of dual inoculation on tissue water, membrane damage and chlorophyll stability*: When inoculated with the microbial consortia, a significant improvement in plant physiological parameters under both the stress periods was observed. Accordingly, relative water content, a measure of plant tissue water status, was higher in dual inoculated plants compared to un-inoculated plants during both the stress periods (Fig. 2a). A study by Barzana et al. [20] revealed that, tomato roots inoculated with AM fungi significantly enhanced the relative apoplastic water flow as compared to non-AM plants and the presence of AM fungi in the roots of host plants was able to modulate the switching between apoplastic and cell-to-cell water transport pathways which maintains leaf relative water content.

Decreased cell membrane damage with increased cell viability was observed in dual inoculated plants compared to un-inoculated plants at both the stress periods (Fig. 2b, c). Therefore, it appears that, dual inoculation seems to provide proper protection to the plants under stress and hence, the  cell membrane damage is less in inoculated plants. Protecting cell membranes and cell viability against moisture stress are crucial as stress can cause disruption of cell membranes and increase the cell solute leakage rate resulting in cell lysis. In drought-stressed soils, the negative water potential poses a challenge for plants to absorb sufficient water. However, AM fungal-mediated aquaporins (AQPs) are vital in osmoregulation and help to overcome this situation [21]. Aroca et al. [22] found upregulation of AQPs contributing to the host plant tolerance to moisture stress when inoculated with *Rhizophagus irregularis* suggesting its primary involvement in host plant tolerance in response to drought. AM fungi inducing changes in various AQP gene expression in the host plants to strengthen root hydraulic conductivity and host tolerance to stress conditions is also documented in many plant species like maize [23], tomato [24], olive [25] and orange [26]. Therefore, maintaining cell membrane integrity is essential for proper cell functioning as it regulates the influx and efflux of water and other solutes into and out of the cell. This process enables cells to remain viable and withstand the effects of stress. A study conducted on Mung bean and French bean [27, 28] under stress conditions reported that, the plants when inoculated with rhizobia and AM fungi showed improved cell membrane integrity. This improvement can be attributed to the plants’ effective nutrient and water uptake mediated by the inoculated AM fungi [29].

Dual inoculation showed an increase in chlorophyll status as analysed by SPAD meter (Additional file 2: Fig. S2a), and the total chlorophyll content quantified spectrophotometrically (Additional file 2: Fig. S2a). Further, the dual inoculated plants also showed a significantly higher chlorophyll stability index (Additional file 2: Fig. S2a) especially under stress conditions, suggesting that, the dual inoculation protect the chlorophyll pigment from degradation. In inoculated plants, *B. liaoningense*, which is an N-fixer might have played a major role in fixing and supplying the required nitrogen resulting in increased chlorophyll content [30]. Wu et al. [31] observed an increased chlorophyll status (SCMR) in populus flowers under moisture stress when inoculated with AM fungi. Similarly, studies on castor bean plants showed increased chlorophyll content (28%) due to *Rhizophagus irregularis* colonization [32]. Therefore, the results unequivocally proved that the dual inoculation protects the chlorophyll from degradation under stress condition.

A higher chlorophyll content and stability index indicates that the chlorophyll is more stable and less susceptible to degradation, which can indicate overall plant's health and stress tolerance. Therefore, the fact that the chlorophyll status readings were higher in the dual inoculated plants compared to the un-inoculated plants suggests that the inoculation may have also led to an increase in the stability of chlorophyll content in the plants.

**Fig. 3:**
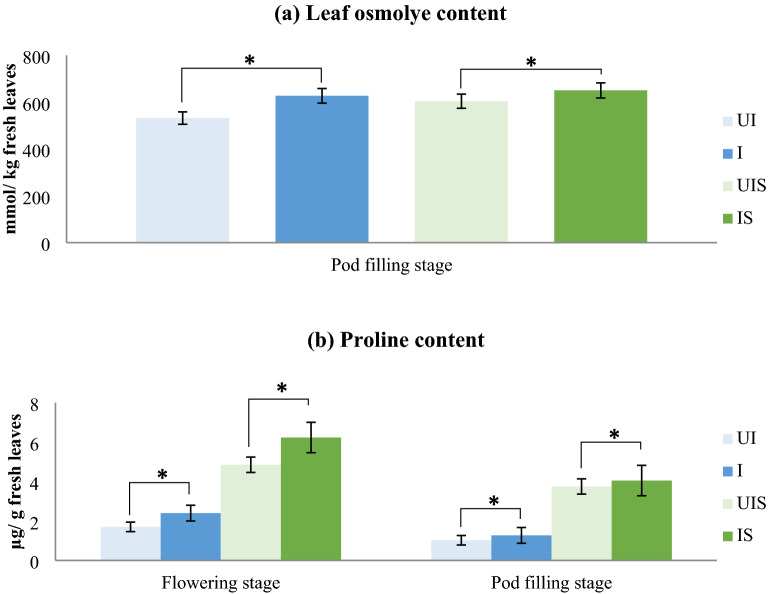
Influence of dual inoculation on **a** leaf osmolyte content and **b** proline content of leaves at flowering and pod filling stage of a drought susceptible soybean cultivar, MAUS 2 grown under irrigated and moisture stressed field conditions. Dual inoculation: *Ambispora leptoticha* + *Bradyrhizobium liaoningense*; *UI* un-inoculated, *I* inoculated, *UIS* un-inoculated stress, *IS*  inoculated stress, Flowering stage: 1st stress period (35–60 DAS), Pod filling stage: 2nd stress period (85–100 DAS); Significant differences (p ≤ 0.05) relative to controls UI & UIS to their respective treatments I & IS are indicated by asterisk (*)

*Influence of dual inoculation on osmolytes accumulation and effective scavenging of reactive oxygen species* (*ROS*): Compatible osmolytes accumulation and reducing the root water potential is one of the strategies adopted by the plants under stress conditions. In the present study, the inoculated plants had accumulated more solutes in the leaves than un-inoculated plants (Fig. 3). Leaf osmolyte content showed increased solutes in the plants under stress conditions in dual inoculated plants compared to un-inoculated plants (Fig. 3a). Even under control condition, the inoculated plants had more solutes in the leaves than un-inoculated plants. It appears that dual inoculation helped the plants to acquire more water by reducing the leaf water potential through the accumulation of compatible solutes. As the soil dries, root cells accumulate a large quantum of compatible solutes/ osmolytes thereby dropping the root water potential to facilitate absorption of available water from the drying soil. Osmolyte accumulation helps in protecting the cellular components which sustain the physiological activity of plants even under stress [33]. In the present study, both *Bradyrhizobium* and AM fungal inoculation effectively regulated osmolytes in plants especially under moisture stress condition [34].

Proline, an amino acid and an important osmolyte is reported to increase several folds due to consequence of drought stress [35, 36]. Accordingly, in the dual inoculated plants, proline accumulation was significantly high compared to un-inoculated plants at both the stress periods (Fig. 3b). Further, the proline content was significantly high in stressed plants compared to irrigated plants. The increased proline levels in inoculated plants might have been triggered due to increased amino acid concentration, because of enhanced N supply by inoculated rhizobia present in root nodules [37]. Kohl et al. [38] have observed higher amounts of proline in soybean plants inoculated with *Bradyrhizobium japonicum* supporting our findings.

**Fig. 4:**
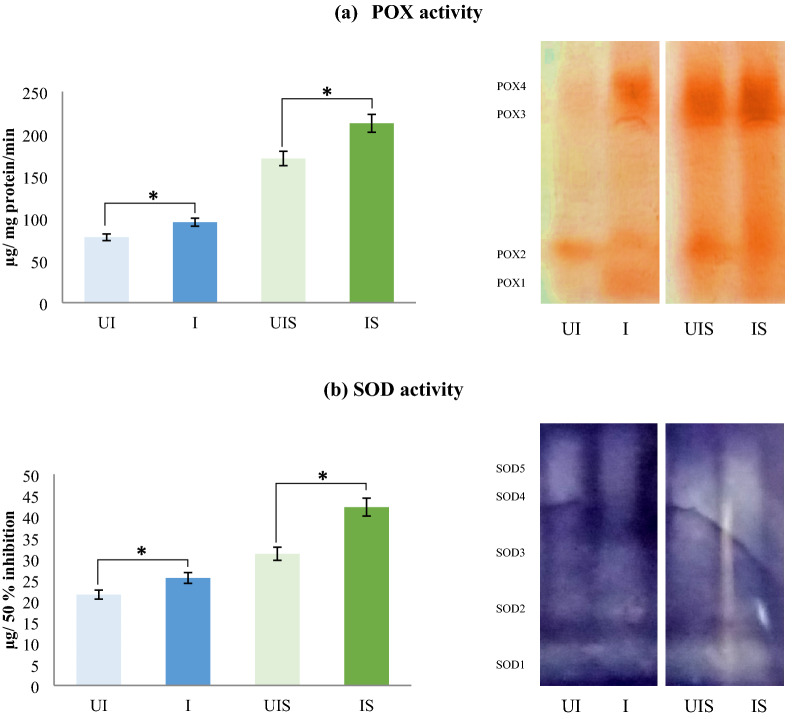
Influence of dual inoculation on **a** guaiacol peroxidase (POX) activity and **b** super oxide dismutase (SOD) activity along with their respective native PAGE assays of leaf samples analysed at pod filling stage of a drought susceptible soybean cultivar, MAUS 2 grown under irrigated and moisture stressed field conditions. Dual inoculation: *Ambispora leptoticha* + *Bradyrhizobium liaoningense*; *UI* un-inoculated, *I* inoculated, *UIS* un-inoculated stress, *IS* inoculated stress, Pod filling stage: 2nd stress period (85–100 DAS); Significant differences (p  ≤ 0.05) relative to controls UI & UIS to their respective treatments I & IS are indicated by asterisk (*)

Drought stress induces the production of free radicals and reactive oxygen species (ROS) which affects cellular functioning leading to oxidative damage [39] and the ultimate death of plants [40]. However, in response to oxidative stress, plants produce ROS scavenging enzymes like catalase, guaiacol peroxidise (POX), superoxide dismutase (SOD), glutathione reductase and other enzymes which help to detoxify the ROS and protect the plants from oxidative damage. In the present field study, POX and SOD were analysed spectrophotometrically followed by native PAGE gel assay (analysed when plants were experiencing moisture stress during pod filling stage). This revealed a high content of POX and SOD enzymes in plants inoculated with microbial consortia compared to un-inoculated plants under both irrigated and stress conditions. Interestingly, the activity of the detoxifying enzymes was significantly more under stress conditions when compared to the control conditions with inoculated plants showing much higher activity (Fig. 4a). This was also evident from the SDA PAGE gel assay where, the POX and SOD iso-enzyme bands were thicker in samples of stressed plants compared to irrigated control plants (Fig. 4b). Within the stress treatment, samples of inoculated plants had visibly thicker bands compared to un-inoculated plants. Other reports also confirm the POX and SOD expression to be at higher levels in AM fungal colonized plants under moisture stress conditions [41]. Bressano et al. [42] have reported that, soybean plants exhibited increased resistance to oxidative stress caused by the herbicide, paraquat when subjected to dual inoculation of AM fungi and rhizobia. Our results therefore, suggest the importance of dual inoculation in reducing the oxidative stress damage to the plants under stress conditions. Various tests conducted in the present study showed that dual inoculation is necessary in soybean to reduce the effects of stress.

**Fig. 5:**
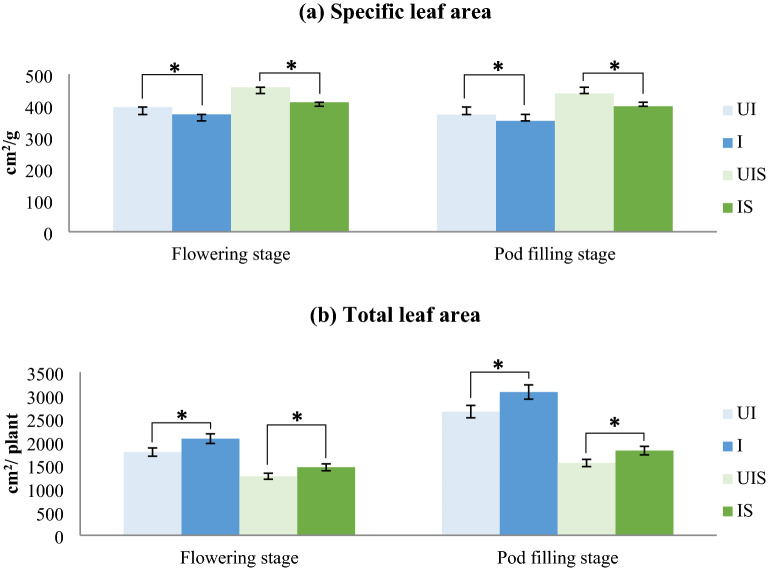
Influence of dual inoculation on **a** specific leaf area and **b** total leaf area analysed at flowering and pod filling stage of a drought susceptible soybean cultivar, MAUS 2 grown under irrigated and moisture stressed field conditions. Dual inoculation: *Ambispora leptoticha* + *Bradyrhizobium liaoningense*; *UI* un-inoculated, *I* inoculated, *UIS* un-inoculated stress, *IS* inoculated stress, Flowering stage: 1st stress period (35–60 DAS), Pod filling stage: 2nd stress period (85–100 DAS); Significant differences (p  ≤ 0.05) relative to controls UI & UIS to their respective treatments I & IS are indicated by asterisk (*)

*Influence of dual inoculation on growth and productivity of soybean*: Several growth and yield parameters were measured in plants grown under different treatments. The specific leaf area (SLA), an indication of leaf thickness was low in dual inoculated plants compared to un-inoculated plants at both the stress periods (Fig. 5a). Furthermore, a lower specific leaf area indicates a thicker leaf and is often associated with greater tolerance to drought. This adaptation strategy can be attributed to the direct influence of AM fungal colonization, which is reported to supply water from deeper layers and nutrients from soil that results in the maintenance of a hydrated state in the plants and thereby, maintain better cellular functioning as compared to those without AM fungal colonization [43]. Total leaf area recorded at both the stress periods revealed that, dual inoculation significantly increased the leaf area in plants grown under both irrigated and stressed conditions with greater increase in irrigated control plants than in stressed plants (Fig. 5b; Additional file 3: Fig. S3). It is evident that, increased leaf area results in increased photosynthesis thereby increasing the plant yield. Increased leaf area under drought stress has been reported earlier in soybean when inoculated with a mixture of *Bradyrhizobium japonicum*, *Rhizophagus intraradices* and *Funneliformis mosseae* [44].

Plant growth parameters like plant height, stem diameter, biovolume index were significantly higher in dual inoculated plants under both irrigated and stressed conditions (Additional file 4: Table S1). AM fungal symbiosis along with bradyrhizobia in improving plant growth in soybean [44] and several other plants [45] is well documented. Such improvement in plant growth and establishment is attributed to the combination of enhanced water and nutrient uptake under moisture stress condition [17, 18]. However, no significant effect of dual inoculation on flowering time was observed between the treatments in the present study as the stress was imposed during the flowering period (Additional file 4: Table S1). The data on flowering depicts that, under stress condition, dual inoculation influenced the early flowering compared to un-inoculated plants. This might be due to improved nutrient and moisture supply, resulting in the plants initiating the next growth and development stages. In fact, several studies in the past have reported that, late or early flowering under drought conditions is specific to crop plants and the growth and developmental phases of the crop at which the stress was imposed [46, 47].

Dual inoculation resulted in increased shoot, root and total dry biomass in inoculated plants compared to un-inoculated plants both under irrigated and stress condition (Table 1). The results also showed a greater root-to-shoot ratio in plants under moisture stress conditions which is a drought escape strategy by the plants to adapt to stress environment [48]. Takacs et al. [44] have reported increased plant dry biomass in soybean due to *Bradyrhizobium japonicum* + *Rhizophagus intraradices* + *Funneliformis mosseae* under drought stress conditions. Similar findings has also been reported earlier in soybean inoculated with *F. mosseae* + *Bradyrhizobium japonicum* [13]. Plants inoculated with microbial consortia had significantly more root volume (nearly 25%) compared to un-inoculated control plants (Table 1). Increased root volume can be correlated to nodulation by rhizobia and a greater soil exploration by root system of host plants colonized by mycorrhizal fungi resulting in increased water and nutrient absorption capacity [45, 49]. This upholds the findings of Sharp et al. [48] who reported increased root volume due to rhizobial nodulation and AM fungal colonization which benefit plants in withstanding moisture stress.

**Table 1:** Influence of dual inoculation on total leaf area, root volume, shoot, root and total dry biomass of a drought susceptible soybean cultivar, MAUS 2 grown under irrigated and moisture stressed field conditions

Treatments	Shoot dry biomass (g/plant)	Root dry biomass (g/plant)	Total plant dry biomass (g/plant)	Root volume (cm^3^/plant)
UI	21.17	3.96	25.13	5.80
I	23.93^*^	4.77^*^	28.70^*^	7.35^*^
UIS	15.25	1.64	16.89	3.38
IS	17.72^*^	2.17^*^	19.95^*^	4.23^*^
SD	0.29	0.13	1.12	0.42
LSD	0.57	0.36	2.20	0.83

Significant differences (p  ≤ 0.05) relative to controls UI & UIS to their respective treatments I & IS are indicated by asterisk (*)

*UI* un-inoculated, *IC* inoculated, *UIS* un-inoculated stress, *IS* inoculated stress, Pod filling stage: 2nd stress period (85–100 DAS), *SD* standard deviation, *LSD* least significant difference

Yield parameters responded positively to dual inoculation under irrigated and moisture stress conditions. The number of pods and pod weight per plant increased by 31% and 34%; the number of seeds and seed weight per plant by 21% and 40% in inoculated plants compared to un-inoculated plants under irrigated condition. Under moisture stress conditions, microbial consortia inoculation increased the number of pods and pod weight per plant by 19% and 34% respectively, and the number of seeds and seed weight per plant by 17% and 32% respectively over un-inoculated plants (Table 2). The same trend was also observed in net plot pod yield and net plot seed yield with 28% and 32% increase over un-inoculated control plants respectively and by 25% and 29% respectively over un-inoculated water-stressed plants due to microbial inoculation. Total dry biomass per plot improved by 33% and 38% in inoculated plants over un-inoculated plants under irrigated and drought-stressed plots respectively due to microbial inoculation. The harvest index increased in microbial inoculated plants grown under irrigated and drought-stressed conditions compared to un-inoculated plants (Table 2). A study of Aliasgharzad et al. [50] with soybean under drought stress reported a significant yield increase in plants inoculated with *Bradyrhizobium japonicum*, *Funneliformis mosseae* and *Claroideoglomus etunicatum*. Similarly, Egamberdiyeva et al. [51] also noticed a significant difference in soybean yield because of inoculation with *Bradyrhizobium* sp. under drought conditions. In fact, several earlier reports showed significantly higher yield in mycorrhizal plants subjected to water deficit in tomato [52], cowpea [53], maize [54] and flax [55]. Significant improvement in plant's growth, yield, quality and establishment is attributed to the combination of enhanced water and nutrient uptake by rhizobial nodulation + AM fungus colonization in host plants under moisture stress creating a favourable environment for stressed plants [56, 57].

**Table 2:** Influence of dual inoculation on pod yield, seed yield and net plot yield in a drought susceptible soybean cultivar, MAUS 2 grown under water irrigated and moisture stressed field conditions

Treatments	Number of pods/plant	Pod weight (g/plant)	Net plot pod weight (g)	Number of seeds/plant	Seed weight (g/plant)	Net plot seed weight (g)	Total dry matter (g/plot)	Harvest index (%)	Δ^13^C (Per-mil)
UI	94.63	39.78	7350.00	183.80	20.17	3978.01	6270.86	63	22.25
I	124.32^*^	53.48^*^	9404.95^*^	223.06^*^	29.25^*^	4954.63^*^	8351.69^*^	71*	21.66^*^
UIS	68.31	19.12	4973.84	89.23	14.13	2238.79	3592.40	55	19.55
IS	81.10^*^	25.65^*^	6544.97^*^	105.06^*^	17.48^*^	2897.89^*^	4977.49^*^	58*	19.98^*^
SD	6.62	3.22	542.97	8.30	2.00	179.32	402.52	1.43	0.18
LSD	11.06	5.38	1252.10	13.36	3.97	413.52	928.24	2.31	0.39

Significant differences (p ≤ 0.05) relative to controls UI & UIS to their respective treatments I & IS are indicated by asterisk (*)

*UI* un-inoculated, *IC* inoculated, *UIS* un-inoculated stress, *IS* inoculated stress, Pod filling stage: 2nd stress period (85–100 DAS), *SD* standard deviation, *LSD* least significant difference

*Influence of dual inoculation on WUE*: Water use efficiency (WUE), one of the important drought adaptive traits measured through the carbon isotope discrimination (Δ^13^C) approach showed a significant improvement in inoculated plants compared to un-inoculated plants. Improved WUE is known to increase the yield of several crops. A significant improvement in productivity through enhanced WUE has been demonstrated and is related to total dry biomass [58]. In the present study, in irrigated condition, Δ^13^C value was high in un-inoculated control plants indicating lower WUE. The assimilation rate was significantly improved in microbial consortia inoculated plants which showed lesser Δ^13^C values (Table 2). It appears that, the assimilation rate of microbial consortia inoculated plants was significantly higher and therefore show a lesser Δ^13^C suggesting higher WUE (Table 2). Under stress conditions, plants try to minimize transpiration by closing stomata which also incidentally affect the assimilation rate [59]. However, plants with dual inoculation could behave normally even under stress conditions and continue to accumulate biomass as they seem to be not affected by stress effects. In fact, inoculated plants under stressed conditions showed higher total dry biomass with high WUE suggesting the importance of dual inoculation in alleviating the stress effects. AM fungal symbiosis has been reported to positively mediate photosynthetic rate [60] , leaf stomatal regulation and transpiration rate [61] as compared to non-mycorrhizal plants.

*Effective colonization and nodulation in inoculated plants*: Nodule numbers and nodule weight was more in microbial consortia inoculated plants which validates effective nodulation by *B. liaoningense* inoculation. Mycorrhizal parameters followed a similar trend where mycorrhizal spore numbers in the rhizosphere soil and per cent mycorrhizal root colonization was significantly more in microbial consortia inoculated plants confirming the effective colonization of *A. leptoticha* in inoculated treatments (Additional file 5: Table S2). Mycorrhizal spore numbers was more in inoculated treatment at both the water regimes. Erman et al. [62] inoculated chickpea plants with rhizobia + AM fungi reported increased nodulation and AM fungal colonization in chickpea plants alleviating drought stress and helping the plants in supply of nutrients and water. Babalola et al. [63] recorded improved nodule biomass of soybean with *Funneliformis mosseae* + *Bradyrhizobium japonicum* as efficient pair than *Glomus deserticola* + *Bradyrhizobium japonicum*. Nodule formation is highly affected in leguminous plants during the drought stress at physiological or molecular level which is found to be extremely sensitive but, co-inoculation with AM fungi improves nodule formation and N fixation which is well documented [64]. The importance of rhizosphere microorganisms [10, 65] and AM fungal influence on the rhizosphere population is also well documented [66, 67]. It is also reported that, rhizopshere microorganisms protect drought stressed plants through various strategies such as adjusting plant hormone levels, and producing osmolytes, antioxidants, and humectants [68]. Hence, it is important to analyse the rhizosphere population which depicts multiple roles of AM fungi in ameliorating moisture stress effects on plants. Qualitative analysis of all the microbial populations (bacteria, fungi, actinomycetes, N fixers, P solubilizers and Zn solubilizers) enumerated in the present study were significantly more in the rhizosphere soil of microbial consortia inoculated plants grown under drought stress conditions (Additional file 6: Table S3). A higher microbial population in the rhizosphere region is evident since the region contains more nutrients and metabolites due to root exudates which increase the microbial activity [69]. Quantitative analysis of dehydrogenase activity based on the reduction of 2,3,5-triphenyltetrazolium chloride (TTC) which analyzes the respiration of viable microorganisms was significantly higher in inoculated plants compared to un-inoculated plants (Additional file 6: Table S3). Many of these microbes are directly or indirectly involved in synergistic process with plant growth mechanisms and hence, it is very important for the plants also to have a healthy rhizosphere microbial population. Growth-promoting microbes in the rhizosphere stimulate the plant growth by synthesis of compounds facilitating the uptake of essential nutrient elements and production of plant growth regulators as well as through antagonistic activity towards plant pathogenic organisms and tolerate against abiotic stresses [10, 35].

Based on one-way and two-way AVOVA, the PLFA microbial biomarker groups were analysed and the results revealed that, the inoculated plants with microbial consortia (before or at stress) showed the presence of higher amounts of all the biomarkers over their respective un-inoculated control (Table 3). The PLFA and NLFA fractions were significantly enhanced due to microbial consortia involving the AM fungus *A. leptoticha* when compared to the un-inoculated plants. Irrespective of conditions (at stress or before stress), inoculation has significantly enhanced AM fungal biomarkers. The higher amounts of fungal to bacterial ratio and Gram-negative stress ratios in rhizosphere of inoculated plants indicate that, soils are less stressed due to the high biomass of AM fungi. In general, fungi are assumed to be less sensitive to changes in moisture and temperature than bacteria due to chitinous cell walls [70]. The principal component analysis (PCA) indicates a clear separation of treatments where, PC1 explains 43.1% of variations and PC2 explains13.2% of variations. The variation along the PC1 axis (43.1%) mainly explains Gram-negative stress ratio, AM fungi-PLFA and NLFA and actinomycetes variables. The big ellipse i.e. regular watered inoculated treatment contributed the highest in terms of the effect of treatments where the maximum contribution was made by AM fungal biomarkers, actinomycetes and Gram-negative stress ratio (Additional file 7: Fig. S4a). The small ellipses i.e. uninoculated plants at stress or regular watered had no biomarkers present (Additional file 7: Fig. S4a). When compared to the total variance, amongst all the variables, maximum contribution was made by AM fungal biomarkers (PLFA and NLFA both), actinomycetes followed by bacteria (Gram-negative and positive) (Additional file 7: Fig. S4b). It indicates that, PLFA biomarkers of AM fungi and actinomycetes act as major drivers in discerning the impact of stress in plants.

**Table 3:** Influence of dual inoculation on rhizosphere microbial communities and AM fungal live-biomass analyzed before stress^#^ and at stress^##^ in a drought susceptible soybean cultivar, MAUS 2 grown under irrigated and moisture stressed conditions by phospholipid fatty acid (PLFA) biomarkers and AM fungal biomarker 16:1ω5cis PLFA & NLFA (all values in nmols/g soil)

	Treatments	Gram + ve	Gram−ve	Anaerobes	Eukaryote	Fungi	Actinomycetes	AM fungi (16:1ω5cis) PLFA)	AM fungi (16:1ω5cis) NLFA)	F/B ratio	Gram -ve stress ratio
Before Stress^#^	UI	30.93	28.37	0.87	2.30	1.66	9.92b	2.07	2.91	0.050	1.083
	I	35.5^*^	32.23	1.88^*^	4.54^*^	2.45	10.61^*^	2.13	4.11*	0.061	1.472*
	UIS	30.60	26.40	1.04	2.51	2.47	9.04	1.91	3.17	0.051	3.574
	IS	34.43^*^	32.20	1.55^*^	4.57^*^	2.32	11.32^*^	2.06	4.11*	0.057	4.017*
At stress^##^	UI	36.45	28.49	1.55	2.48	1.63	11.86	2.08	3.62	0.060	3.990b
	I	44.28^*^	37.58	1.86^*^	4.60^*^	2.28	14.88^*^	3.52^*^	12.10*	0.059	4.587*
	UIS	27.90	24.81	1.02	2.38	1.26	6.29	1.77	3.28	0.044	3.185
	IS	28.09	31.89	1.38^*^	3.27^*^	1.74	10.32^*^	2.39^*^	9.92*	0.062	3.832*
	SD	4.77	4.89	0.34	0.76	0.33	1.05	0.23	1.17	0.042	0.148
	LSD	10.11	10.36	0.71	1.61	0.70	2.23	0.49	2.47	0.042	0.148
2-Way ANOVA	Inoculation (I)	^*^	NS	^*^	^*^	NS	^*^	^*^	*	NS	*
	Stress (S)	NS	NS	NS	NS	^*^	NS	^*^	*	NS	*
	Interaction I × S	NS	NS	NS	NS	NS	^*^	^*^	*	NS	*

Significant differences (p  ≤ 0.05) relative to controls UI & UIS to their respective treatments I & IS, and between interactions are indicated by asterisk (*)

*UI* un-inoculated, *IC* inoculated, *UIS* un-inoculated stress, *IS* inoculated stress, Pod filling stage: 2nd stress period (85–100 DAS), *SD* standard deviation; *LSD* least significant difference, *NS* not significant

^#^Analyzed on 34 DAS (before imposing stress), ##Analyzed on 50 DAS (at stress)

*Influence of dual inoculation on plant nutrients status*: Plant macro and micro-nutrients in general, decreased under moisture stressed conditions compared to regular watering. Microbial consortia inoculation significantly improved the nutrient uptake under stress (Table 4). The major nutrients such as N, P and K were significantly higher in inoculated plants which are important for plant's growth to withstand moisture stress. Mg was also higher in inoculated plants which is essential for chlorophyll formation. The role of AM fungi in P supply to plants and its importance in alleviating drought stress in plants is well documented [49]. Increased N is due to inoculated *Bradyrhizobium* through the N-fixation process [71]. Carbon absorption and distribution between plant shoot and root can be affected by an inadequate supply of P which negatively affects the functions and growth of nodules as P is needed for N fixation process for energy transformation in nodules to achieve maximum function [72, 73]. The inoculated AM fungus supplies the required amount of P, which plays a critical role in the nutrient exchange between the host plants and the fungus. Supporting the above results, Aliasgharzad et al. [50] have reported AM fungi with *Bradyrhizobium japonicum* increased plant N and K nutrients compared to un-inoculated soybean plants under pot culture trials.

**Table 4:** Influence of dual inoculation on plant macro nutrients and micro nutrients concentration in a drought susceptible soybean cultivar, MAUS 2 grown under irrigated and moisture stressed field conditions

Treatments	Macro nutrients (%)					Micro nutrients (ppm)					
	N	P	K	Ca	Mg	Zn	Fe	Cu	Mn	B	Mo
UI	1.39	0.10	1.00	2.65	0.98	146.86	4070.0	136.20	148.50	170.80	87.14
I	2.17*	0.18*	1.05*	3.74*	1.35*	166.90*	6753.0*	162.50*	167.90*	215.10*	114.72*
UIS	1.34	0.09	0.75	2.33	0.61	73.41	1169.0	95.30	85.180	135.30	40.97
IS	2.01^*^	0.14^*^	0.92^*^	3.49^*^	0.92^*^	91.26^*^	2712.0^*^	111.00^*^	124.50^*^	155.80^*^	56.54^*^
SD	0.01	0.01	0.01	0.01	0.11	0.82	77.94	0.8	0.82	0.82	0.82
LSD	0.02	0.02	0.02	0.02	0.29	1.88	179.72	1.87	1.88	1.88	1.88

Significant differences (p≤ 0.05) relative to controls UI & UIS to their respective treatments I & IS are indicated by asterisk (*)

*UI* un-inoculated, *IC* inoculated, *UIS* un-inoculated stress, *IS* inoculated stress, Pod filling stage: 2nd stress period (85–100 DAS), *SD* standard deviation, *LSD* least significant difference

The tripartite symbiosis in legumes, rhizobia and AM fungi is well documented and is one of the most important ecological mutualisms where the plants benefit with necessary N through rhizobial symbiosis and P through AM fungal symbiosis [12]. In turn, the rhizobia benefits with necessary P supply to both nodules and plants by AM fungi. The AM fungi in turn, benefits by photo-assimilates supply from the host plant [74]. The microbial consortia comprising of N-fixing bacteria *Bradyrhizobium liaoningense* + P-supplying AM fungi *Ambispora leptiticha* with abilities to supply necessary moisture and nutrients as and when required to the host plants conferred drought tolerance to the drought susceptible and high yielding soybean cultivar, MAUS 2 in the present study. Based on the various data recorded like physiological, biometric, yield, microbial and nutrient parameters, the inoculated microbial consortia in this study alleviated the moisture stress effect on the drought susceptible soybean cultivar, MAUS 2 and minimized the yield loss significantly. Symbiotic N fixation by inoculated rhizobia supplied major N requirement required by the plants at both flowering and pod filling stages and the AM fungi directly and preferentially stimulate the nodule function increasing N supply. Further, AM fungal symbiosis enhanced the plant growth through hyphal-mediated enhanced water uptake leading to enhanced osmotic adjustment in roots, maintaining high leaf water potential, reducing oxidative damage to lipids and supplying P from soil resulting in improved stress tolerance.

In general, a significant difference in physiological, growth and biometric parameters was observed between plants grown with and without dual inoculation. Further, plants grown under stress conditions with dual inoculation showed no significant difference with those grown under irrigated conditions with no inoculation suggesting the importance of dual inoculation in overcoming the stress effects in a drought susceptible soybean cultivar. Thus, the study under field condition revealed that, rhizobia and AM fungal inoculation is necessary for soybean in instances when the crop undergoes severe drought stress and alleviates the stress significantly at multiple growth and developmental stages.

## Conclusion

The present study revealed that, dual inoculation with rhizobia *B. liaoningense* + AM fungus *A. leptoticha* has improved the physiological, biometric, yield, microbial and nutrient parameters of a drought susceptible and high-yielding soybean cultivar, MAUS 2. Under moisture stress conditions, dual inoculation increased the number of pods and pod weight per plant by 19% and 34% respectively; and the number of seeds and seed weight per plant by 17% and 32% respectively over un-inoculated plants. Thus, microbial consortia inoculation helped in nutrient mobilization, and water uptake and alleviated moisture stress in soybean cultivar, MAUS 2. Further, rhizobia plus AM fungal inoculation are necessary for soybean to alleviate drought stress at multiple growth and developmental stages.

## Material and methods

### Location, cropping and treatment details

The experiment was conducted during rabi (winter) season starting from October at the University of Agricultural Sciences,  Bangalore (UASB) situated at an altitude of 920 m from mean sea level. The average maximum and minimum temperature that prevailed during the experimental period was 28 °C and 18 °C respectively, while the sunshine hours were 6.66 h and during the experimental period, a total rainfall of 110 mm was received. However, with the protective gear (protective cover/ canopy), moisture stress was imposed at the flowering and pod filling stages (incidentally, no rains received during the stress imposition period). The site where the experiment was conducted was used for raising finger millet crop prior to conduct of this experiment and was left fallow for three months. The cropping and treatment details are given under Additional file 8: Table S4.

Soil in the study area is classified as uniform, red sandy loam indicating consistent temperature regimes and the presence of kaolinite clay minerals that contribute to its fertility. Soil samples collected from this area was analysed for various physico-chemical properties and the data is presented below. The soil pH was 6.2 determined using digital pH meter, while, the electrical conductivity analysed with the help of the Conductivity Bridge measured 0.11 dS/m. Soil organic carbon and available N, P and K was 0.45%, 259.94; 28.40 and 207.61 kg/ha respectively which were estimated following the standard procedures [75].

Native AM fungal spore population was 20 spores/g of soil and the rhizobial population was not observed as evidenced from serial dilution plating method using YEM agar medium estimated following standard procedures [76]. The physiological and microbial parameters were analysed at UASB, Bangalore and at the Centre for Natural Biological Resources and Community Development (CNBRCD), Bangalore respectively.

### Soybean variety

The experiment was performed with a high yielding and drought susceptible soybean cultivar, MAUS 2 [Germplasm Accession No. MAUS 2; UASB]. The cultivar was selected based on an earlier field experiment conducted to investigate the drought tolerance abilities along with various other varieties (unpublished data). The shortlisted cultivars were then screened with different AM fungi [76] followed by another drought adaptive trials with single and dual inoculation of selected rhizobia and AM fungus conducted at greenhouse conditions [77]. Later, a micro-plot experiment was conducted with drought susceptible cultivar, MAUS 2 and a drought tolerant cultivar, DSR 12 [Germplasm Accession No. HARDEE; ICAR-IISR, Indore] to study their stress response through inoculation with microbial consortia (rhizobia and AM fungi) [19].

### *Ambispora leptoticha* inoculum

The AM fungus, *Ambispora leptoticha* being an obligate symbiont was multiplied in plastic pots using the substrate mix containing vermiculite, perlite and soilrite in the ratio of 3:1:1 v/v/v under glasshouse conditions and Rhodes grass (*Chloris gayana* Kunth) as the host. After 75 days of growth, shoots of Rhodes grass were severed and the substrate containing spores, hyphae and root bits (cut into about 1 cm pieces) was air dried and used as the inoculum. The infective propagule (IP) numbers of the AM fungus was estimated by the most probable number (MPN) method [78] and was found to be 1700 IP/g of inoculum.

### *Bradyrhizobium liaoningense* inoculum

*B. liaoningense* sp. nov. (MTCC 10753/NCBI No. JF792426) was procured from Indian Council of Agricultural Research-Indian Institute of Soybean Research (ICAR-IISR), Indore, India. *Bradyrhizobium liaoningense* was used in this experiment as it was proven to be the best rhizobial species for improving growth of soybean [79]. The procured culture was sub-cultured on yeast extract mannitol (YEM) agar medium with Congo red and incubated at 28 °C for 5 days. Pure isolated single colonies were picked and inoculated to YEM broth and incubated at 28 °C for 5 days. Fully grown broth culture was cold centrifuged and the bacterial pellets collected was mixed in dilute phosphate buffer and used as inoculum. The rhizobial population of the inoculum mixed in phosphate buffer was estimated by serial dilution and plating method which had 1 × 10^9^ CFU/ml of inoculum. The inoculum was added to 10% starch solution (1:1 v/v) and this inoculum mixture was mixed with soybean seeds to form a seed coat. The coated seeds were shade dried and sown. The population of *B. liaoningense* coated on the seeds was enumerated by serial dilution method and was found to be 1 × 10^6^ CFU/seed.

### Experimental setup

The study plot was prepared, brought to a fine tilth and farmyard manure (FYM) was applied and mixed thoroughly with soil. Using randomized block design, plots with four different treatments having 4 replications were made. Chemical fertilizers (procured from Zuari Agro Chemicals Ltd, Bangalore) i.e. nitrogen in the form of urea (46% N) @ 66.66 kg/ha, phosphorus in the form of SSP (16% P_2_O_5_) @ 500 kg/ha and potassium in the form of MOP (60% K_2_O) @ 63.33 kg/ha were applied in seeding rows.

*Ambispora leptoticha* was added @ 10 g/plant evenly in the furrows made in the plots according to the details of the treatments [76]. Control plots received only the substrate vermiculite, soilrite and perlite (3:1:1 v/v/v basis). *B. liaoningense* coated seeds of soybean cultivar, MAUS 2 were sown (3 seeds/ seeding point, later thinned to leave 1 seedling) in furrows. Control plots were sown with seeds coated with starch. Plots were irrigated after sowing. Later, protective irrigation was given once every 3 days. T1 and T2 plots were irrigated till harvest while, moisture stress was imposed on T3 and T4 treatments at 2 different developmental stages of the crop growth period. Irrigation was stopped and moisture stress was imposed at the early reproductive phase for 25 days when the plants start flowering from 35 to 60 days after sowing (DAS) and re-irrigated from 61 to 84 DAS to alleviate the stress and to allow the plants to recover from stress effects. Moisture stress was imposed again for 15 days from 85 to 100 DAS when the plants were at the pod filling stage. Later, these plots were irrigated till maturity and harvest. In a nutshell, the crop was imposed with moisture stress at two crucial developmental stages namely, flowering and pod filling stages to examine whether, the microbial consortia have any role in overcoming the stress effect and thus, imparting stress tolerance to the soybean plants.

All the physiological and biometric parameters described below were analysed at both the stress periods (35–60 DAS and 85–100 DAS). However, leaf osmotic concentration, peroxidase and superoxide dismutase enzyme activity were analysed during1st stress period between 35 and 60 DAS and the water use efficiency by carbon isotope discrimination (Δ^13^C) approach was analysed during 2nd stress period between 85 and 100 DAS. The parameters analysed following the standard methodologies as described by earlier workers [19, 77, 80, 81].

### Measurement of physiological parameters

#### Soil physical parameters

Soil physical parameters viz. soil moisture, soil temperature and soil water potential were analysed during both the stress periods (35–60 and 85–100 DAS). The soil sample from a depth of 0–10 cm was collected from each plot and brought to laboratory for analysis. Soil moisture was estimated by gravimetric method using the formula.

$$Soil \,moisture\, (\%)=\frac{Wt. \,of \,moist \,soil -Wt. \,of \,dried \,soil}{Wt. \,of \,dried \,soil} \times 100$$

Soil temperature (^o^C) and soil water potential (ψ) was measured using an Dew point potentiameter device (WP4 Dewpoint Potentiometer manufactured by Decagon Devices, Inc., USA).

#### Relative water content

Relative water content (RWC) was measured by analysing fresh weight (FW), turgid weight (TW) and dry weight (DW) of leaves. The following formula was used to determine RWC [80, 81].

$$RWC \, (\%)=\frac{FW-DW}{TW-DW} \times 100$$

#### Cell membrane damage

Cell membrane damage was analysed as outlined by Leopold et al. [82] by measuring the intensity of solutes leaked out of the leaf cells. The leaf samples of known weight were incubated in water for 3 h in a glass beaker and the leachate (L0) was measured at 273 nm in a spectrophotometer (Spectronic 20D + by Thermo Scientific, USA). Later, the leaves were boiled for 15 min at 100 °C and the leachate (L1) absorbance was recorded. The percent leakage was calculated as per the formula given below.

$$Solute \,leakage (\mathrm{\%})\hspace{0.17em}=\hspace{0.17em}[(L0) / (L1)] \times 100$$

#### Cell viability

Cell viability was measured by 2,3,5-triphenyl tetrazolium chloride (TTC) reduction test [83]. The viability of the tissue is reflected as colour formation when TTC is reduced to red formazon in living and respiring tissues. The intensity of the colour formation was measured by recording the absorbance at 485 nm in spectrophotometer (Spectronic 20D + by Thermo Scientific, USA). The absorbance values indicate the direct reflection of leaf cell viability.

#### Leaf chlorophyll status (SPAD value, total chlorophyll content and chlorophyll stability index)

Using a portable SPAD (Soil Plant Analysis Development) meter (Minolta Corp., Ramsey), a non-destructive analysis of chlorophyll/ nitrogen  status in the leaf was measured by clamping the SPAD meter onto the leaf at different positions as well as on different leaves. The mean of several SPAD Chlorophyll Meter Readings (SCMR) for each treatment was calculated and presented as unit less SCMR value. Using UV–Visible spectrophotometer (SpectraMax Plus 384 by Molecular Devices, USA), the total chlorophyll content (TCC) and chlorophyll stability index (CSI) were determined following the modified method of Hiscox and Israelstam [84]. The values obtained were then substituted in the below-mentioned equation to determine total chlorophyll content (TCC) and chlorophyll stability index (CSI).

$$Total \,chlorophyll \,content \, (mg/g \,fresh\, wt. \,leaf)\hspace{0.17em}=\hspace{0.17em}(A 652/ 34.5)\hspace{0.17em}\times \hspace{0.17em}(V/ Fresh \,Weight)$$

$$CSI\hspace{0.17em}=\hspace{0.17em}100 -R$$

Where, $$R\hspace{0.17em}=\hspace{0.17em}[(control-stressed)/control]\hspace{0.17em}\times \hspace{0.17em}100$$

(Note: A-Absorbance, V-Volume of acetone and DMSO solution)

#### Leaf osmolyte content

Leaf samples from 5 plants from all the plots were collected, wrapped in aluminium foil and frozen in liquid nitrogen. The samples were then thawed and centrifuged for 5 min at 12,000 rpm and the extracted sap was collected. The extracted sap was measured for leaf osmolyte content using VAPRO vapour pressure osmometer (WescorInc, Logan, USA).

#### Proline estimation

Proline increases proportionately under water stress faster than other amino acids in plants and is one of the important osmolytes accumulated under stress which makes the plants to absorb water even under low soil water status by keeping the root water potential lower than the soil. The analysis was carried out based on the procedure given by Bates [85].

#### Assessing the activity of ROS detoxifying enzymes

Stress generally induces the production of free radicals and ROS damaging the membrane system. In response to this, plants also have a defensive mechanism where through the production of detoxifying enzymes, they could control the damage caused by ROS and other toxic compounds. In this study, the defensive  enzymes such as POX and SOD were analysed in the samples collected during 2nd stress period (85–100 DAS) coinciding with pod filling stage to examine whether or not the dual inoculation has any role in regulating the stress effects through the defensive mechanism.

*Protein extraction*: Plant leaf samples from all the treatments were collected and frozen using liquid nitrogen to prevent proteolytic activity before being homogenized using a pestle and mortar and the homogenate was suspended in extraction buffer [Phosphate buffer 0.1 M, pH 7.8, 1 mM PMSF (protease inhibitor) and 0.1% of polyvinylpyrollidon (PVP)] in an Eppendorf tube and kept on ice for 15 min. The crude protein extract was centrifuged at 14,000 rpm at 4 °C for 30 min in a cooling centrifuge. Later, the pellet was discarded and the soluble protein supernatant was used for further analysis. Protein concentration was determined by Lowry’s method [86] using bovine serum albumin (BSA) as standard.

*Enzyme assays*: POX enzyme activity in the protein extract was measured by the method proposed by Castillo et al. [87] with slight modification. Peroxidase activity was assayed as the increase in optical density due to the oxidation of guaiacol to tetra-guaiacol. Native PAGE was performed as per the method described by Davis [88] for peroxidase isoenzyme activity using 10% resolving gel and 5% stacking gel. Protein extract (25 μg) of all the treatments were loaded to the gel separately. Gel electrophoresis was run initially at 80 V and later, once protein entered the resolving gel, increased to 120 V. Electrophoresis was conducted at 40 °C for about 3 h. Later, the gel was stained for peroxidase isoenzymes.

SOD activity was measured by the method described by Dhindsa et al. [89] with slight modifications. SOD activity in the supernatant was assayed by its ability to inhibit photochemical reduction of nitroblue tetrazolium. Native PAGE was performed according to the method described by Davis [88] for superoxide dismutase isoenzyme activity using 5% stacking gel and 10% resolving gel. Protein extract (25 μg) from all the sample treatments were loaded to gel. Electrophoresis was performed initially at 60 V and after the protein entered the resolving gel, the voltage was increased to 120. The electrophoresis was conducted for 3 h at 40 °C. The gel was incubated in a staining solution containing 100% NBT (nitroblue tetrazolium chloride) (w/v), 0.2 M EDTA (Ethylenediaminetetraacetic acid) (w/v), 0.1 M sodium phosphate buffer (pH 7.5), commercial grade TEMED (Thermo Scientific Pierce Tetramethylethylenediamine) and 5% riboflavin (w/v) for 30 min until the bands appeared. The isoenzyme bands appeared as white/colourless in a dark blue background and the isoenzyme pattern was photographed.

### Measurement of growth and yield attributes

#### Specific leaf area

Specific leaf area (SLA), an expression of leaf thickness was determined as per the formula given below [80, 81].

$$SLA \left(cm^{2}/mg \,dry \,leaf \,per \,plant\right)=\frac{Leaf \,area }{Leaf \,weight}$$

#### Total leaf area

Total leaf area (TLA) was recorded at two stress periods and at harvest by measuring the specific leaf area of 5 randomly selected leaves. WinDIAS 3 Image Analysis System [90] and electronic weighing balance were used to measure leaf area and the leaf dry weight respectively. Later, at harvest, all the leaves were separated and collected from the plant, oven dried and multiplied with SLA to get the total leaf area.

#### Plant height, stem diameter, biovolume index and days to 50% flowering

The plant growth parameters like plant height and stem diameter were determined before harvest. Plant height was recorded from the soil surface to the growing tip of the plant using a measuring scale and the stem diameter was measured just above the soil surface using digital Vernier Callipers. Biovolume index (BI) was calculated by the formula given by Hatchell et al. [91]. The plant growth parameters were also recorded during the stress period (35–60 DAS) and before harvest; however, only the final plant growth parameter readings recorded at harvest are presented. Plants were monitored regularly for the appearance of the first flower. The number of days taken from sowing to flowering was recorded.

#### Plant dry biomass and root volume

After harvest, plants’ shoot and root parts were separated from 20 sampled plants and collected in paper bags individually. The plant material was dried in a hot air oven (manufactured by Servewell Instruments Pvt. Ltd., Bangalore) at 60 °C to a constant weight. Later, the weight of shoot and root of each replication was weighed on a standard weighing balance. Total dry biomass of plant was calculated by summing up both shoot and root biomass. In order to determine the net plot biomass and yield, all the plants in the plot were uprooted, plant (shoot + root) and pods separated, and dried. The net plot biomass from each plot and yield was noted. Root volume per plant was determined by the water displacement method outlined by Harrington et al. [92]. The root samples separated from harvested plants were submerged in a graduated beaker containing known amount of water. The volume of water displaced when root is submerged in water was measured and expressed as cm^3^ per plant.

#### Yield and harvest index

Pods from 20 randomly selected plants were collected at maturity and individual plant pod yield was recorded. Later, the seeds were separated from the pod and the number of seeds/ plant and weight of seeds/ plant were recorded. All the plants grown in each plot were harvested to determine the net plot pod and seed weight. Harvest index (HI), a measure of reproductive efficiency was calculated by using the formula given by Donald and Hamblin [93].

$$HI = \left(Seed \,yield\div Total \,dry \,matter\right)\times 100$$

### Determination of WUE based on carbon isotope discrimination (Δ^13^C) technique

In nature, there exists two stable isotopes of carbon viz., ^12^C and ^13^C of which, major carbon share is ^12^C with 98.9% and rest 1.1% is ^13^C. Overall, ^13^C abundance relative to ^12^C in the plant is less common. The Δ^13^C in plant samples was determined using a sophisticated analytical instrument called Isotope Ratio Mass Spectrometer (IRMS). The plant sample is converted to CO_2_ by combustion and the isotopic composition is determined. Leaf samples collected to measure SLA were subjected to carbon isotope discrimination analysis [80, 81]. The leaf samples were dried and finely powdered with a metal beads and 1 mg of finely powdered sample was then taken in silver capsules and crimped and placed sequentially in the carousel of the auto-sampler. The samples were then dropped at precise times along with an injection of pure O_2_ into the oxidation reactor. Once the sample combustion takes place inside the instrument, the instrument records the C discrimination values (Δ^13^C values). The analysis to determine the carbon isotope discrimination using IRMS was done at a National Facility for Stable Isotope Studies in Biological Sciences installed at the Department of Crop Physiology, UAS, Bangalore.

### Microbial parameters

#### Rhizobial and mycorrhizal parameters

Rhizobial nodule numbers were counted manually and the weight was recorded from 20 randomly selected plants under each treatment at harvest. The mycorrhizal parameters like per-cent root colonization was carried out as per the procedure proposed by Philips and Hayman [94] and spore numbers in the root zone soil was determined by wet sieving and decantation procedure as outlined by Gerdemann and Nicolson [95].

#### Enumeration of rhizosphere beneficial microflora

The rhizosphere samples were collected at harvest from 5 plants from all the replications under each treatment in a polythene cover and immediately brought to the laboratory and stored at − 20 °C for further analysis. The samples during analysis were pooled, and taken as representative composite samples under each treatment. The total bacterial, fungal, actinomycetes and N-fixers population were determined using spread plate technique using Soil Extract Agar, Martin’s Rose Bengal Agar, Kenknight and Munaier’s Agar, Combined Carbon Agar medium respectively [96].

#### Dehydrogenase activity in rhizosphere soil

Dehydrogenase activity in soil serves as an indicator of microbial oxidative activities and is a measure of total microbial activity. The activity was determined by the procedure given by Casida et al. [97] using 2, 3, 5-triphenyl tetrazolium chloride (TTC). The microbial activity is reflected as colour formation when TTC is reduced to red formazon and intensity of colour formation was measured by recording the absorbance at 485 nm in a spectrophotometer (Spectronic 20D + by Thermo Scientific, USA).

#### Phospholipid fatty acid extraction (PLFA) and quantification

PLFA analysis was carried out on column chromatography and lipids were analysed on gas chromatograph with flame ionization detector (GC-FID). PLFA biomarkers specific to microbial community including AM fungi were quantified. PLFA were analysed as per the method described by Buyer and Sasser [98] & Sharma and Buyer [99]. About 2 g of freeze-dried rhizosphere soil samples were used to extract the lipids by solid-phase extraction (SPE) followed by extraction of phospholipids by SPE. The chloroform fraction from the SPE was used for neutral lipid fatty acids (NLFA) analysis while, the 5:5:1 (chloroform: methanol: water) fraction was used for PLFA analysis. NLFA and PLFA fractions were converted to fatty acid methyl esters by transesterification and analysed by gas chromatography. The PLFAs were summed into biomarker categories as follows: gram-positive bacteria, Gram-negative bacteria, anaerobe, eukaryote, fungi, actinomycetes, AM fungi (PLFA), AM fungi (NLFA). FAME profiles of PLFA were identified using the MIDI PLFAD1 calibration mix and peak naming table (MIDI, Inc., DE, USA) and from NLFA run, only profile of 16:1ω5cis was used representing AM fungal biomass.

### Macro- and micro-nutrients in plant tissue

The plant N, P and K concentration was estimated following the Micro-kjeldahl method, vanadomolybdate phosphoric yellow colour method and flame photometer method respectively [75]. The plant Ca and Mg concentrations were estimated by EDTA titration method [75]. The plant micro nutrients Zn, Fe, Cu, Mn and Bo were estimated by atomic absorption spectroscopy [100].

### Statistical analysis

Raw data of each parameter were subjected to one-way analysis of variance (ANOVA) at a significance level of 5% and means were compared by Duncan’s multiple range test (DMRT) when F-values were significant using Costat statistical software (Costat/Cohort statistical software, CA, USA) and AgRes Statistical Software (Ver. 3.01) by Pascal Intl. Software Solutions and SAS Institute Inc. [101]. Microbial fatty biomarkers data were subjected to Principal Component Analysis (PCA) to determine the main contributing factors to the total variance across the stress and inoculation treatments. PCA was performed in R version 3.6.0 by using R packages, ‘factoextra’, ‘FactoMineR’, ‘devtools’ and ‘ggbiplot’ [98, 99].

## Supplementary Information

**Additional file 1: Fig. S1**. Influence of dual inoculation on **a** soil moisture, **b** soil water potential and **c** soil temperature of study plots analyzed at flowering and pod filling stage in a drought susceptible soybean cultivar, MAUS 2 grown under irrigated and moisture stressed field conditions. Dual inoculation: *Ambispora leptoticha* + *Bradyrhizobium liaoningense*; *UI* un-inoculated, *I* inoculated, *UIS* un-inoculated stress, *IS* inoculated stress, Flowering stage: 1st stress period (35-60 DAS); Pod filling stage: 2nd stress period (85-100 DAS); No significant differences (p ≤ 0.05) observed relative to controls UI & UIS and their respective treatments.

**Additional file 2: Fig. S2**. Influence of dual inoculation on **a** SPAD chlorophyll meter reading (SCMR) value, **b** Total chlorophyll content and **c** Chlorophyll stability index of leaves at flowering and pod filling stage in a drought susceptible soybean cultivar, MAUS 2 grown under irrigated and moisture stressed field conditions. Dual inoculation: Ambispora leptoticha + Bradyrhizobium liaoningense; *UI* un-inoculated, *I* inoculated, *UIS* un-inoculated stress, *IS* inoculated stress, Flowering stage: 1st stress period (35-60 DAS), Pod filling stage: 2nd stress period (85-100 DAS); Significant differences (p ≤ 0.05) relative to controls UI & UIS are indicated by asterisk (*) & hash (#) respectively.

**Additional file 3: Fig. S3**. Comparison of leaf growth in soybean cultivar, MAUS 2 inoculated with *Ambispora leptoticha + Bradyrhizobium liaoningense *grown under irrigated and moisture stressed field conditions.

**Additional file 4: Table S1**. Influence of dual inoculation on plant height, stem diameter, biovolume index and flowering in a drought susceptible soybean cultivar, MAUS 2 grown under irrigated and moisture stressed field conditions.

**Additional file 5: Table S2**. Influence of dual inoculation on number of nodules, nodule weight, mycorrhizal spore numbers in the root zone soil and the percent mycorrhizal root colonization in a drought susceptible soybean cultivar, MAUS 2 grown under irrigated and moisture stressed field conditions.

**Additional file 6: Table S3**. Influence of dual inoculation on rhizosphere microbial communities and dehydrogenase activity before and after imposing the stress in a drought susceptible soybean cultivar, MAUS 2 grown under irrigated and moisture stressed field conditions

**Additional file 7: Fig. S4**. **a** Principal Component analysis of the PLFA biomarkers from the rhizosphere soils of soybean cultivar, MAUS 2 inoculated with *Ambispora leptoticha* + *Bradyrhizobium liaoningense*. **b** PLFA variables subjected to redundancy analysis by PCA. [10 PLFA biomarkers (including 2 ratios) were selected for the PCA model where axis labels indicate variables that were strong negative or positive factors on each axis (biomarker- Fungi, Gram −ve, Eukaryote contributed negatively]; *UI* un-inoculated, *I* inoculated, *UIS* un-inoculated stress, *IS* inoculated stress.

**Additional file 8: Table S4**. Summary of cropping and treatment details.

## Data Availability

The datasets used and/or analysed during the current study are available from the corresponding author on reasonable request.
