# Supplementary material for: Ameliorating the drought stress tolerance of a susceptible soybean cultivar, MAUS 2 through dual inoculation with selected rhizobia and AM fungus
Source: Fungal Biol Biotechnol. 2023 May 3;10:10. doi: 10.1186/s40694-023-00157-y (PMC10158380; doi:10.1186/s40694-023-00157-y)
Supplement: Supplementary file 2 — Additional file 2: Fig. S2. Influence of dual inoculation on a SPAD chlorophyll meter reading (SCMR) value, b Total chlorophyll content and c Chlorophyll stability index of leaves at flowering and pod filling stage in a drought susceptible soybean cultivar, MAUS 2 grown under irrigated and moisture stressed field conditions. Dual inoculation: Ambispora leptoticha + Bradyrhizobium liaoningense; UI un-inoculated, I inoculated, UIS un-inoculated stress, IS inoculated stress, Flowering stage: 1st stress period (35-60 DAS), Pod filling stage: 2nd stress period (85-100 DAS); Significant differences (p ≤ 0.05) relative to controls UI & UIS are indicated by asterisk (*) & hash (#) respectively. [file 40694_2023_157_MOESM2_ESM.docx]

*****

*****

*****

*****

*****

*****

*****

*****

*****

*****

*****

Additional file 2: Fig. S2: Influence of dual inoculation on a) SPAD chlorophyll meter reading (SCMR) value, b) Total chlorophyll content and c) Chlorophyll stability index of leaves at flowering and pod filling stage in a drought susceptible soybean cultivar, MAUS 2 grown under irrigated and moisture stressed field conditions. Dual inoculation: *Ambispora leptoticha* + *Bradyrhizobium liaoningense*; UI= Un-inoculated; I= Inoculated; UIS= Un-inoculated stress; IS= Inoculated stress; Flowering stage: 1^st^ stress period (35-60 DAS); Pod filling stage: 2^nd^ stress period (85-100 DAS); Significant differences (p ≤ 0.05) relative to controls UI & UIS for (a) & (b) to their respective treatments I & IS are indicated by asterisk (*), and UI & I for c) to their respective treatments UIS & IS are indicated by asterisk (*).
