# Supplementary material for: Ameliorating the drought stress tolerance of a susceptible soybean cultivar, MAUS 2 through dual inoculation with selected rhizobia and AM fungus
Source: Fungal Biol Biotechnol. 2023 May 3;10:10. doi: 10.1186/s40694-023-00157-y (PMC10158380; doi:10.1186/s40694-023-00157-y)
Supplement: Supplementary file 3 — Additional file 3: Fig. S3. Comparison of leaf growth in soybean cultivar, MAUS 2 inoculated with Ambispora leptoticha + Bradyrhizobium liaoningense grown under irrigated and moisture stressed field conditions. [file 40694_2023_157_MOESM3_ESM.docx]

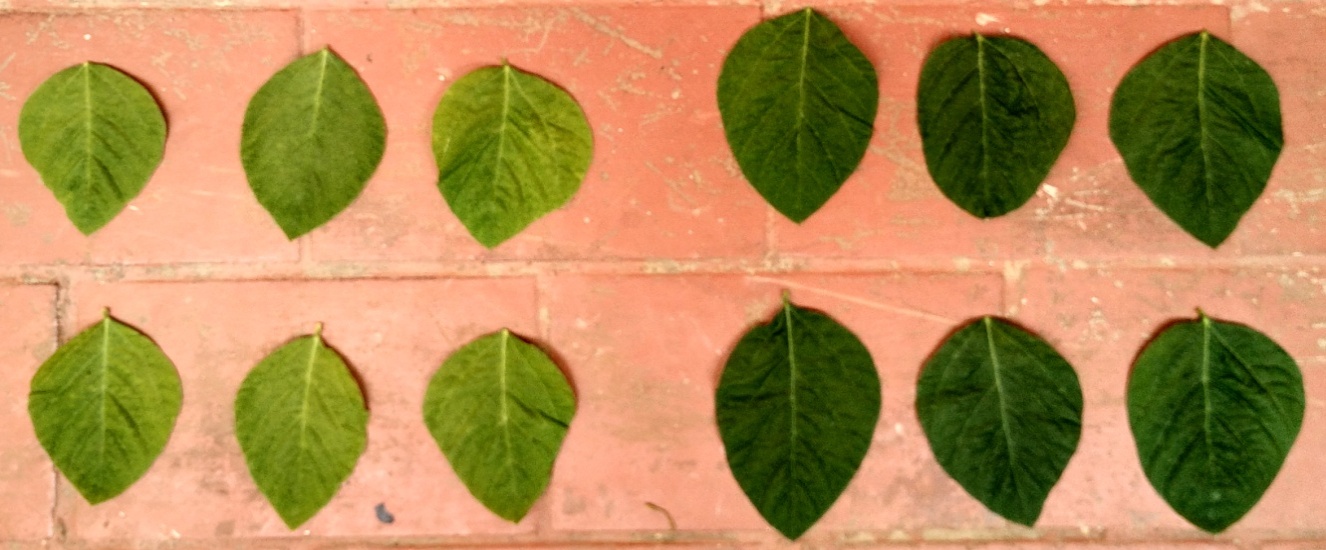


Irrigated un-inoculated control Irrigated inoculated control


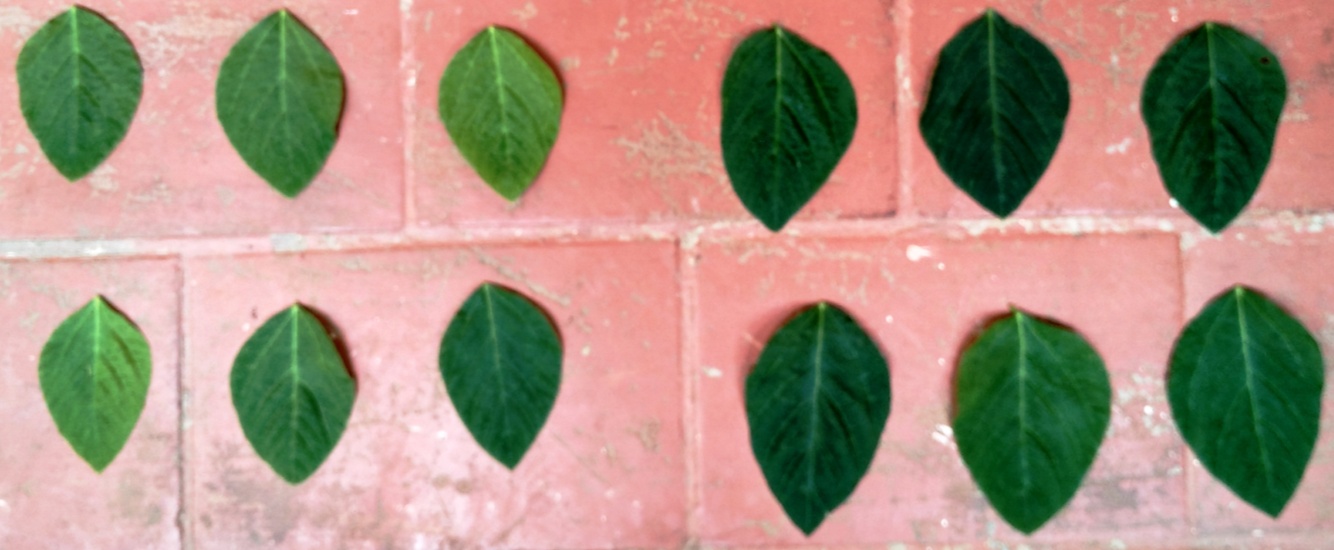


Un-inoculated stress Inoculated stress

Additional file 3: Fig. S3: Comparison of leaf growth in soybean cultivar, MAUS 2 inoculated with *Ambispora leptoticha + Bradyrhizobium liaoningense* grown under irrigated and moisture stressed field conditions.
