# Supplementary material for: Ameliorating the drought stress tolerance of a susceptible soybean cultivar, MAUS 2 through dual inoculation with selected rhizobia and AM fungus
Source: Fungal Biol Biotechnol. 2023 May 3;10:10. doi: 10.1186/s40694-023-00157-y (PMC10158380; doi:10.1186/s40694-023-00157-y)
Supplement: Supplementary file 4 — Additional file 4: Table S1. Influence of dual inoculation on plant height, stem diameter, biovolume index and flowering in a drought susceptible soybean cultivar, MAUS 2 grown under irrigated and moisture stressed field conditions. [file 40694_2023_157_MOESM4_ESM.docx]

Additional file 4: Table S1: Influence of dual inoculation on plant height, stem diameter, biovolume index and flowering in a drought susceptible soybean cultivar, MAUS 2 grown under irrigated and moisture stressed field conditions.

| Treatments | Plant height  (cm/ plant) | Stem dia.  (mm/ plant) | Biovolume  Index | Days to 50 %  Flowering |
| --- | --- | --- | --- | --- |
| UI | 32.16 | 8.43 | 271.16 | 40 |
| I | 32.25* | 9.39* | 302.90* | 39* |
| UIS | 22.78 | 6.14 | 142.20 | 37 |
| IS | 24.08* | 6.96* | 167.65* | 38* |
| SD | 0.76 | 0.56 | 17.30 | NS |
| LSD | 1.52 | 1.07 | 32.49 |  |

UI= Un-inoculated; IC= Inoculated; UIS= Un-inoculated stress; IS= Inoculated stress; Pod filling stage: 2^nd^ stress period (85-100 DAS); SD: Standard error of deviation; LSD: Least significant difference; Significant differences (p ≤ 0.05) relative to controls UI & UIS to their respective treatments I & IS are indicated by asterisk (*); NS = Not significant.
