# Supplementary material for: Ameliorating the drought stress tolerance of a susceptible soybean cultivar, MAUS 2 through dual inoculation with selected rhizobia and AM fungus
Source: Fungal Biol Biotechnol. 2023 May 3;10:10. doi: 10.1186/s40694-023-00157-y (PMC10158380; doi:10.1186/s40694-023-00157-y)
Supplement: Supplementary file 5 — Additional file 5: Table S2. Influence of dual inoculation on number of nodules, nodule weight, mycorrhizal spore numbers in the root zone soil and the percent mycorrhizal root colonization in a drought susceptible soybean cultivar, MAUS 2 grown under irrigated and moisture stressed field conditions. [file 40694_2023_157_MOESM5_ESM.docx]

Additional file 5: Table S2: Influence of dual inoculation on number of nodules, nodule weight, mycorrhizal spore numbers in the root zone soil and the percent mycorrhizal root colonization in a drought susceptible soybean cultivar, MAUS 2 grown under irrigated and moisture stressed field conditions.

| Treatments | Nodule nos./  plant | Nodule wt.  (g/ plant) | Mycorrhizal spore  nos./ 50 g soil | Mycorrhizal  colonization (%) |
| --- | --- | --- | --- | --- |
| UI | 08 | 0.06 | 60 | 71 |
| I | 91* | 3.86* | 165* | 88* |
| UIS | 07 | 0.06 | 36 | 55 |
| IS | 82* | 2.03* | 156* | 70* |
| SD | 1.22 | 0.21 | 12.26 | 6.32 |
| LSD | 3.58 | 0.98 | 28.27 | 3.19 |

UI= Un-inoculated; IC= Inoculated; UIS= Un-inoculated stress; IS= Inoculated stress; Pod filling stage: 2^nd^ stress period (85-100 DAS); SD: Standard error of deviation; LSD: Least significant difference; Significant differences (p ≤ 0.05) relative to controls UI & UIS to their respective treatments I & IS are indicated by asterisk (*)
