# Supplementary material for: Ameliorating the drought stress tolerance of a susceptible soybean cultivar, MAUS 2 through dual inoculation with selected rhizobia and AM fungus
Source: Fungal Biol Biotechnol. 2023 May 3;10:10. doi: 10.1186/s40694-023-00157-y (PMC10158380; doi:10.1186/s40694-023-00157-y)
Supplement: Supplementary file 6 — Additional file 6: Table S3. Influence of dual inoculation on rhizosphere microbial communities and dehydrogenase activity before and after imposing the stress in a drought susceptible soybean cultivar, MAUS 2 grown under irrigated and moisture stressed field conditions [file 40694_2023_157_MOESM6_ESM.docx]

Additional file 6: Table S3: Influence of dual inoculation on rhizosphere microbial communities and dehydrogenase activity before and after imposing the stress in a drought susceptible soybean cultivar, MAUS 2 grown under irrigated and moisture stressed field conditions

|  | Treatments | CFU/ g of rhizosphere sample | | | | | | Dehydrogenase activity  (µg of TPF released/  g soil/ h) |
| --- | --- | --- | --- | --- | --- | --- | --- | --- |
|  |  | Bacteria  (10^5^) | Fungi  (10^3^) | Actinomycetes  (10^5^) | N fixers  (10^4^) | P solubilizers  (10^3^) | Zn solubilizers  (10^3^) |  |
| Before stress^^^ | UI | 140 | 120 | 300 | 53 | 20 | 170 | 1350 |
|  | I | 300* | 310* | 450* | 62* | 80* | 200* | 4950* |
|  | UIS | 150 | 115 | 350 | 48 | 15 | 160 | 1650 |
|  | IS | 350* | 330* | 400* | 63* | 74* | 210* | 4150* |
| At stress^^ | UI | 170 | 220 | 500 | 33 | 21 | 40 | 2900 |
|  | I | 350* | 450* | 560* | 80* | 110* | 90* | 4650* |
|  | UIS | 90 | 270 | 540 | 30 | 10 | 10 | 1250 |
|  | IS | 100* | 420* | 600* | 65* | 34* | 30* | 3450* |
|  | SD | 0.96 | 3.07 | 1.71 | 0.82 | 1.71 | 1.08 | 58.35 |
|  | LSD | 2.03 | 6.51 | 3.62 | 1.73 | 3.62 | 2.30 | 123.70 |

UI= Un-inoculated; IC= Inoculated; UIS= Un-inoculated stress; ^Analyzed on 34 days after sowing; ^^Analyzed on 50 days after sowing; Significant differences (p ≤ 0.05) relative to controls UI & UIS to their respective treatments I & IS are indicated by asterisk (*); SD: Standard error of deviation; LSD: Least significant difference
