# Supplementary material for: Ameliorating the drought stress tolerance of a susceptible soybean cultivar, MAUS 2 through dual inoculation with selected rhizobia and AM fungus
Source: Fungal Biol Biotechnol. 2023 May 3;10:10. doi: 10.1186/s40694-023-00157-y (PMC10158380; doi:10.1186/s40694-023-00157-y)
Supplement: Supplementary file 7 — Additional file 7: Fig. S4. a Principal Component analysis of the PLFA biomarkers from the rhizosphere soils of soybean cultivar, MAUS 2 inoculated with Ambispora leptoticha + Bradyrhizobium liaoningense. b PLFA variables subjected to redundancy analysis by PCA. [10 PLFA biomarkers (including 2 ratios) were selected for the PCA model where axis labels indicate variables that were strong negative or positive factors on each axis (biomarker- Fungi, Gram −ve, Eukaryote contributed negatively]; UI un-inoculated, I inoculated, UIS un-inoculated stress, IS inoculated stress. [file 40694_2023_157_MOESM7_ESM.docx]

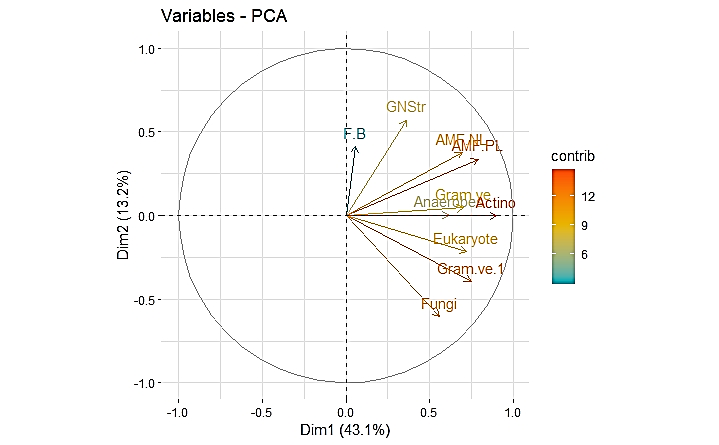

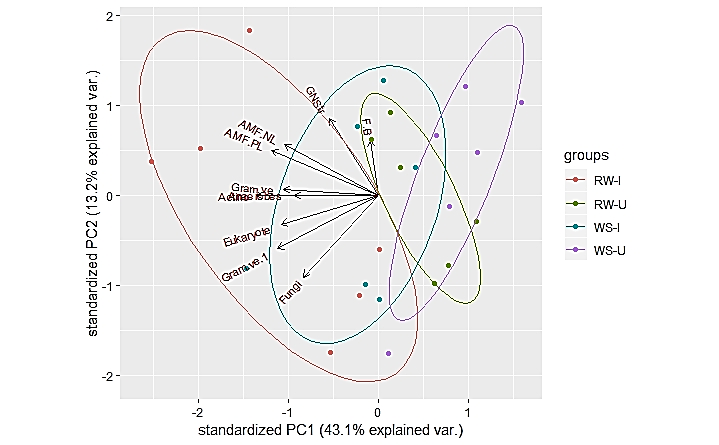


a

b

**UI**

**I**

**UIS**

**IS**

Additional file 7: Fig. S4: a) Principal Component analysis of the PLFA biomarkers from the rhizosphere soils of soybean cultivar, MAUS 2 inoculated with *Ambispora leptoticha* + *Bradyrhizobium liaoningense*. b) PLFA variables subjected to redundancy analysis by PCA. [10 PLFA biomarkers (including 2 ratios) were selected for the PCA model where axis labels indicate variables that were strong negative or positive factors on each axis (biomarker- Fungi, Gram –ve, Eukaryote contributed negatively]; UI= Un-inoculated; I= Inoculated; UIS= Un-inoculated stress; IS= Inoculated stress
