# Supplementary material for: Ameliorating the drought stress tolerance of a susceptible soybean cultivar, MAUS 2 through dual inoculation with selected rhizobia and AM fungus
Source: Fungal Biol Biotechnol. 2023 May 3;10:10. doi: 10.1186/s40694-023-00157-y (PMC10158380; doi:10.1186/s40694-023-00157-y)
Supplement: Supplementary file 8 — Additional file 8: Table S4. Summary of cropping and treatment details. [file 40694_2023_157_MOESM8_ESM.docx]

Additional file 8: Table S4: Summary of cropping and treatment details.

| Crop | Soybean |
| --- | --- |
| Cultivars | MAUS2 (drought susceptible) |
| Season | Rabi (Winter) |
| Temperature; humidity; precipitation | 28 ^o^C to 18 ^o^C; 65 to 74%; 110 mm |
| Recommended dose of fertilizer (RDF) | 30:80:38 kg of N: P_2_O5: K_2_O/ ha |
| Farm Yard Manure (FYM) | 6.25 T/ha |
| Plot design & size | Randomized Block Design; |
| Plot size & spacing | 4m × 3m plot size and spacing between rows was 30 cm and between plants was 20 cm (200 plants/plot) |
| Treatment details | T1: Un-inoculated irrigated  T2: Inoculated irrigated  T3: Un-inoculated stress  T4: Inoculated stress |
